# Updating Expectations About Unexpected Object Motion in Infants Later Diagnosed with Autism Spectrum Disorder

**DOI:** 10.1007/s10803-021-04876-2

**Published:** 2021-01-30

**Authors:** Sheila Achermann, Terje Falck-Ytter, Sven Bölte, Pär Nyström

**Affiliations:** 1 Development and Neurodiversity Lab, Department of Psychology, Uppsala University, Box 1225, 75142 Uppsala, Sweden; 2 Center of Neurodevelopmental Disorders (KIND), Centre for Psychiatry Research, Department of Women’s and Children’s Health, Karolinska Institutet, Stockholm, Sweden; 3 Swedish Collegium for Advanced Study (SCAS), Uppsala, Sweden; 4 Curtin Autism Research Group, School of Occupational Therapy, Social Work and Speech Pathology, Curtin University, Perth, WA USA

**Keywords:** Infants, Autism spectrum disorder, Visual motion, Prediction, Eye tracking, Tolerance for uncertainty

## Abstract

In typical development, infants form predictions about future events based on incoming sensory information, which is essential for perception and goal-directed action. It has been suggested that individuals with autism spectrum disorder (ASD) make predictions differently compared to neurotypical individuals. We investigated how infants who later received an ASD diagnosis and neurotypical infants react to temporarily occluded moving objects that violate initial expectations about object motion. Our results indicate that infants regardless of clinical outcome react similarly to unexpected object motion patterns, both in terms of gaze shift latencies and pupillary responses. These findings indicate that the ability to update representations about such regularities in light of new information may not differ between typically developing infants and those with later ASD.

**Supplementary Information:**

The online version of this article (10.1007/s10803-021-04876-2) contains supplementary material, which is available to authorized users.

## Introduction

Acting efficiently in an ever changing, dynamic environment requires individuals to actively make predictions about upcoming events. From early on, infants learn to perceive and understand others’ actions and the regularities of objects in the world surrounding them (Gredeback et al. 2002; von Hofsten 2004), and to adapt their predictions based on experiences about them accordingly. One way to explore infants’ ability to form such expectations about future events is to use moving objects. A widely used paradigm to investigate visual motion prediction is to study infants’ representation of moving and temporarily hidden (occluded) objects (Aguiar and Baillargeon 1999; Baillargeon 1999; Kochukhova and Gredeback 2007; Meltzoff and Moore 1998; Moore et al. 1978; Rosander and von Hofsten 2004; Spelke et al. 1994; von Hofsten et al. 2000). Already at around 12 weeks of age, infants begin to form expectations about how objects move in time and space, and track objects even if they are temporarily occluded (Rosander and von Hofsten 2004).

Recently, different information processing accounts of autism spectrum disorder (ASD) have been put forward that all underline the importance of prediction, and the adaptation of predictions to changes in the environment. It is assumed that humans constantly make predictions about the environment and these predictions are integrated and compared with incoming sensory information to form a percept, which is needed to be able to act efficiently in the world (Pellicano and Burr 2012). There are different theories suggesting different parts of prediction as the driving factor for atypical perception and cognition often observed in ASD. For instance, it has been suggested that autistic individuals are less biased by their prior experience when forming percepts. Consequently, making perceptions in ASD might be biased towards bottom-up processing rather than top-down processing, leading to “more realistic” percepts (Pellicano and Burr 2012). Interestingly however, probing the robustness of long-term priors in ASD, Croydon et al. (2017) found that both autistic and neurotypical children showed the “light-from-above” bias when interpreting shading patterns. Another theory proposes that autistic individuals assign atypically high weight to prediction errors, which impacts perceptual processing (the HIPPEA model: High, Inflexible Precision of Prediction Errors in Autism; Van de Cruys et al. 2014). High weighting of the precision of prediction errors causes predictive models to be updated more frequently, and because the system is supposed to be inflexible, the weighting is not adjusted to different environments as much as in neurotypical individuals. High precision of prediction errors is not a disadvantage in all environments, but it is thought that in the inherently imprecise *social* world, it can lead to over-fitting and a sense of uncontrollability (Van de Cruys et al. 2014). Importantly, such deviations in prediction and adaptation should affect our cognitive systems at all levels, including basic perception of motion. Some studies have found that autistic children show reduced adaptation/malleability of priors in certain occasions (e.g., time perception: Karaminis et al. 2016; adaptation to loudness: Lawson et al. 2015), while others found adaptation in ASD to be no different than neurotypical individuals (e.g., motion prediction: Tewolde et al. 2018; Mooney images: Van de Cruys et al. 2017).

When viewing an ambiguous stimulus, Turi et al. (2018) found that perceptual processing, measured by the pupillary response, was correlated with participants’ Autism Quotient Score (AQ: Baron-Cohen et al. 2001). The results were interpreted as indication of a different perceptual processing style in an ambiguous situation related to autistic traits. However, Laeng et al. (2018) found that autistic and neurotypical individuals showed similar pupillary responses to illusions.

ASD symptoms emerge early in life; hence if prediction problems are fundamental in the condition, they should be present before or at the same time as the emergence of symptoms. However, to our knowledge, there are no studies that have investigated early manifestations of prediction alterations in relation to later ASD symptoms.

The current study followed infants at elevated likelihood for ASD (with older full biological siblings diagnosed with ASD) and infants at low likelihood for ASD (with older full biological undiagnosed siblings) from 10 to 36 months of age, when a clinical diagnostic assessment was performed. The recurrence rate of ASD in full siblings is around 20% on average (Constantino et al. 2010; Ozonoff et al. 2011).

Using an eye tracking task at 10, 14 and 18 months of age, we investigated how the infants performed gaze shifts towards the reappearance location of a temporarily occluded object, allowing us to test their expectations about the future trajectory of moving objects and how expectations are updated in light of new information. The object changed direction behind the occluder, making the location of reoccurrence unexpected at first, because the default prediction is that objects move along a linear path (Kochukhova and Gredeback 2007). Thus, across trials, we could assess how infants adapted to this unexpected motion trajectory. In addition to gaze shifts, we also measured pupil size. Greater pupil dilation has been observed when infants are presented with surprising, physically impossible events (Jackson and Sirois 2009) as well as irrational social events (Gredeback and Melinder 2010). Greater pupil dilation is related to an increase in arousal and cognitive load (Laeng et al. 2012), and is closely linked to the locus coeruleus norepinephrine system in the brain (Aston-Jones and Cohen 2005). Thus, we explored the pupillary response to the reappearance of a temporarily occluded object and a sudden change in object motion to investigate infants’ reaction to a violation of their expectation.

In order to investigate infants’ ability to update their expectations based on new visual information, we examined both gaze shift latencies and pupillary responses. We expected that infants regardless of diagnostic outcome would show adaptation over trials (indicated by faster gaze shifts towards the target and the pupil size becoming less affected by the occlusion passage). Secondly, we investigated potential differences in adaptation rates in the 10–18 months range between infants with later ASD and neurotypical infants, taking into account the potential effect of age. In addition, we specifically analyzed the gaze shift and pupillary response to the first trial (because the events observed in the first trial are unexpected in relation to prior experience outside the experiment). Here too, we examined potential differences between infants with a subsequent diagnosis of ASD and neurotypically developing infants in terms of gaze shift latency and change in pupil size in response to the first occlusion trial across ages. Given the notion that individuals with ASD have an atypical balance between bottom-up and top-down processes, which is essential for forming predictions in new situations, we expected group differences in this task. However, to our knowledge, no one has studied the update of expectations in the current context of infants with later ASD, we refrained from making specific predictions about the direction of the putative effects.

## Methods

### Participants

After exclusion, the final sample included 91 infants, with data from assessments performed at 10, 14, and 18 months of age (see section *Assessment at 10, 14 and 18 months of age* for the amount of valid gaze data per assessment and Table 1 for group comparisons of background characteristics). All participants were part of the Early Autism Sweden study (EASE; for a general overview, see http://www.earlyautism.se). The EASE study is a prospective longitudinal study of infant siblings at elevated likelihood of ASD. At the time of enrollment, the clinical outcome is unknown; thus, infants with an older sibling diagnosed with ASD represent the “elevated likelihood” group, while infant siblings with no familial history of ASD represent the “low likelihood” group. At 36 months of age, a clinical diagnostic assessment was conducted and children were assigned to (1) an elevated likelihood group with ASD (EL-ASD); (2) an elevated likelihood group without ASD (EL-no-ASD); or (3) a low likelihood group with neurotypical outcome (LL), with the characteristics summarized in Table 1.

**Table 1:** Participant characteristics by group, final samples (*n, Mean, SD*) at 10, 14 and 18 months of age

Measure	LL (29, 14 females)			EL-no-ASD (47, 31 females)			EL-ASD (14, 6 females)			Bayesian ANOVA
	*n*	*Mean*	*SD*	*n*	*Mean*	*SD*	*n*	*Mean*	*SD*	*BF*_*01*_^a^
Age 10 m (days)	29	310.5	14.49	48	312.0	12.39	14	310.2	16.91	7.86
Age 14 m (days)	28	431.7	21.36	46	432.2	17.18	13	437.6	10.53	5.93
Age 18 m (days)	27	558.7	26.05	45	557.4	17.83	13	556.7	16.63	8.16
SES^b^, education	29	4.5	0.99	48	4.2	1.2	14	3.9	1.3	3.24
MSEL^c^, at 10 m	29	99.9	12.2	48	101.9	14.3	14	97.9	10.4	5.69

^a^*BF*_*01*_ describes the likelihood of the data under *H*_*0,*_* BF*_*01*_ = 3–10, moderate evidence for *H*_*0*_

^b^SES, Socioeconomic status, based on parental education on a five level rank scale

^c^MSEL, Mullen scales of early learning, early learning composite score

Participating families were recruited through multiple channels, including the project’s website, advertisements, clinical units, and a database of families within the larger Stockholm area who had indicated interest in research participation previously. All children included in the study were born at full term (> 36 weeks) and no infant had any confirmed or suspected medical problems (including visual and auditory impairments).

All families provided written informed consent and the study was approved by the Regional Ethical Board in Stockholm. The study was conducted in accordance with the standards specified in the 1964 Declaration of Helsinki.

### Assessment at 10, 14 and 18 months of age

Participants in the EASE study undergo multiple measures and assessments including eye tracking (Falck-Ytter et al. 2018; Nyström et al. 2018, 2019), motion tracking (Achermann et al. 2020), electroencephalography (EEG), magnetic resonance imaging (MRI), parent–child interaction, and developmental assessments, spending 4–5 h in the lab. This study includes data from the eye tracking session and the developmental assessment using the Mullen Scales of Early Learning (MSEL; Mullen, 1995), at 10, 14 and 18 months of age. Regarding the eye tracking session, at 10 months of age 76.8% of infants provided valid gaze data (*n* = 70), at 14 month of age 70.0% provided valid gaze data (*n* = 63, 1 infant did not complete the assessment), and at 18 months of age 80.5% of infants provided valid gaze data (*n* = 70, 4 infants did not complete the assessment).

### Assessment at 36 months of age

At 36 months, a clinical diagnostic assessment was conducted by experienced psychologists and included standardized information on medical history, current developmental, and adaptive level, as well as autistic symptoms using the Autism Diagnostic Interview-Revised (ADI-R; Rutter et al. 2003), the Autism Diagnostic Observation Schedule 2nd Edition (ADOS-2; Lord et al. 2012), and the Mullen Scales of Early Learning (MSEL; Mullen 1995).

### Data collection

The current eye tracking task was part of a larger eye tracking session (total duration of approximately 8 min) including other experiments (Falck-Ytter et al. 2018; Kleberg et al. 2018) which are not relevant for the current research questions and which are not analyzed here. The eye tracking session typically took place after the lunch break, and a nap if the infant was tired. The infants were seated on their parent’s lap at approximately 60 cm distance to the computer monitor where the stimuli were presented. Gaze data were collected using Tobii corneal reflection eye trackers (Tobii AB, Danderyd, Sweden). During this longitudinal study, equipment changes occurred, such that data were first recorded on a Tobii 1750 at 50 Hz and after an upgrade with a Tobii TX300 eye tracker at 120 Hz. The two trackers used different sampling rates and screen resolutions, but after temporal upsampling using linear interpolation and spatial resampling (stimuli were presented at different screen resolutions but the same physical size), all recordings displayed a resolution of 1024 × 768 pixels and a screen size of 23″ with a sample rate of 300 Hz. Before the eye tracking session, the eye tracker was calibrated using a five-point calibration procedure in which a coloured sphere expanded and contracted on the screen in synchrony with sound. The sphere expanded sequentially at five locations on the screen (i.e., every corner and the centre). The procedure was repeated if necessary, until an acceptable calibration of both eyes was obtained (as in Kleberg et al. 2018; Nyström et al. 2018).

### Stimulus

**Fig. 1:**
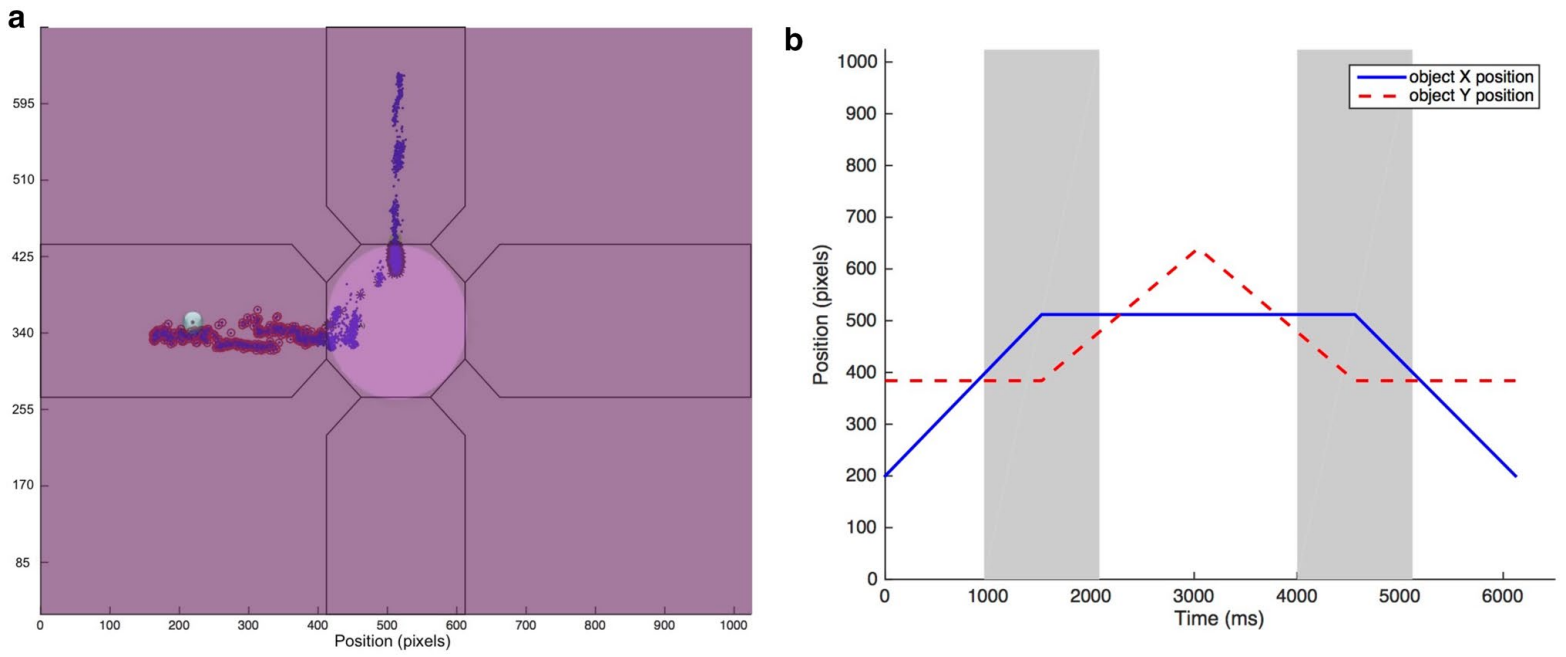
**a** Two-dimensional gaze data plotted in blue and superimposed on the visual scene during the experiment. Illustration of the areas of interest (AOI) covered by the analysis during an example trial. The octagon AOI in the middle of the screen covered the occluder. The target object (the ball) can be seen to the left in this example. **b** The object’s X- and Y-position separately plotted over time during an example trial with two occlusion intervals colored in grey

The stimulus consisted of a moving object (a ball; radius = 10 px, 0.44°; Fig. 1) that started moving horizontally from the left side of the screen with constant speed, accompanied by a music track to increase interest in the task. In the middle of the screen, after 960 ms, the moving object disappeared behind a circular occluder (radius = 100 px, 4.36°). At the center of the screen (behind the occluder) the object changed direction 90° counter-clockwise, and continued moving in this direction. The object reappeared after 1120 ms, and continued upward for 960 ms. Then, the object reversed the direction and moved back to the starting point along the same trajectory. Thus, the moving object rolled back and forth between two endpoints at constant velocity in a horizontally flipped L-shape (Fig. 1). For analysis, we defined one trial as one occlusion passage from one endpoint to the other (3040 ms), either starting on the side and ending on top, or starting on top and ending on the side. Each infant was presented with 2 blocks consisting of 10 such occlusion passages; hence, every session included 20 occlusion passages (trials). Within the blocks, the trials formed a continuous back-and-fort movement of the ball following its L-shaped trajectory. A subgroup of infants saw 3 blocks of the task due to changes in the experimental setting; however, this additional block was excluded from the analysis in order to create congruent preconditions for all infants included in the study.

To assess gaze data, five areas of interest (AOIs) were defined. These included four oblong rectangles for each possible direction of the moving object and one AOI for the occluder. The size of each AOI was kept constant throughout the experiment (see Fig. 1).

### Data reduction

Raw gaze coordinates were analyzed in MATLAB (R2015a, Mathworks Inc., CA, USA) using custom written scripts. First, we measured the time the object was completely occluded and time the object started to reappear again. These timestamps were then used to define a window of interest for the analysis. Occlusion always occurred 1120 ms before reappearance, and the former was defined as time point 0. Trials with less than 50% gaze data prior the event (i.e., reappearance) were automatically discarded. Infants contributing with less than 4 trials were excluded from the analysis (*n* = 19). Next, we interpolated gaze data linearly over gaps shorter than 15 samples (i.e. 50 ms). All trials underwent visual inspection, blinded for infant identity and group status, in order to remove trials containing movement artefacts, noisy data, or missing data close to the occlusion event. Visual inspection was done by plotting the two-dimensional gaze data over time and included the AOIs for the trajectory of the moving object and the occluder (see Supplementary Figures). Gaze data were manually transposed so that the AOIs covered as much gaze as possible and accounted for gaze calibration drifts during the recording. Then, gaze velocity was calculated and plotted in order to identify the gaze shift latency towards an AOI after occlusion.

The pupillary response to the reappearance of the moving object after temporary occlusion was measured after the gaze shift towards the object. The change in size of the pupil was calculated based on a 2000 ms time interval after the gaze shift relative to a baseline measure prior to the gaze shift (1000 ms), and converted a percentage (i.e. 100 is same size as baseline, < 100 means pupil constriction, and > 100 means pupil dilation). All pupil measurements were taken from the left eye instead of the average of both eyes to avoid artifacts if the eye tracker lost track of one of the eyes.

### Dependent variables

The behavioral measures of the study were gaze shift latencies and pupillary responses as described above, which were measured both across trials within each testing session and across the three different time points (10, 14, and 18 months). Individual trial values were used as the most basic dependent variables, and performance on the first trial was of particular interest. In a first step, we were interested in general effects in the entire sample, regardless of group and age. Bayesian one sample *t*-tests against 0 including the average adaptation rate and the first trial response were conducted in order to detect general effects regarding gaze shift latency and pupillary responses.

**Fig. 2:**
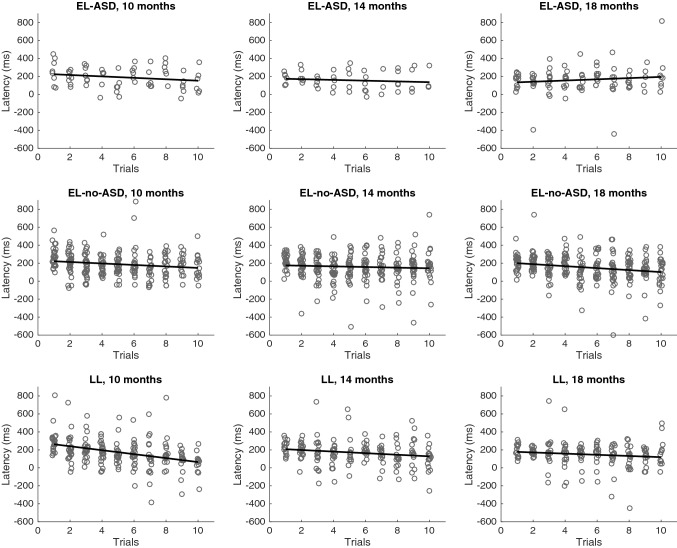
Gaze shift latency to the reappearing object over trials for all groups (EL-ASD, EL-no-ASD, and LL) and ages. Circles represent trial latency from individual infants for transparency purposes. Note that the statistical analyses were performed on the *individual trial slopes* (termed *adaptation rate*), and the *slope of adaptation rate or first trial value across ages* (termed *developmental change*), whereas the black line correspond to the linear regression of the data in the plot which does not account for repeated measures within individuals

Next, because the focus of the study is predictive and adaptive behavior, the dependent variables included the *adaptation rates* across trials for gaze shift latency and pupillary responses. Adaptation rate was operationalized as the slope of a linear regression within each participant with at least 4 valid trials (as an example see Fig. 2, where the trial responses across trials for gaze shift latencies are shown, and the adaptation rate on a group level is represented by the regression line). There were no significant differences between block 1 and 2 in terms of trial means, or adaptation rates within blocks. Therefore, in order to include infants who had spuriously excluded trials in one of the block, the two blocks were averaged to increase the number of data points for the individual adaptation rates.

To investigate age differences, we calculated a similar linear regression slope across ages (10, 14, and 18 months) for each infant and measure, which is hereafter, termed *developmental change.* The developmental change was calculated both for the first trial values and for adaptation rates.

The approach of testing regression slopes (adaptation rates and developmental change) instead of performing repeated measures analyses, makes it possible to include participants which had missing data for one of the time points. Testing the slope between groups is similar to testing the interaction effect between age and group in a repeated measures ANOVA, but does allow for missing data. An option would be to use Linear Mixed Models (LMMs), but because we use Bayesian statistics (see our motivation below) our approach provides a mathematically easier solution than using Bayesian LMMs. It is not possible to test for “main effects” of group when comparing slopes, but testing the average values across ages provides equivalent results. Together, the first trial values (similar to an intercept), the slope (adaptation rate and developmental change), and the average across ages, give thorough information about the collected data. For completeness, we also show data from specific ages.

In the results section we present all statistical tests using the subsections (1) first trial gaze shift latency, (2) adaptation rate of gaze shift latency, (3) first trial pupil response, and (4) adaptation rate of pupil response.

### Statistical analysis

The data was analyzed using Bayesian *t*-tests and ANOVAs implemented in JASP (JASP Team 2019, Version 0.9.0.1). The support for our hypotheses is described by the Bayes factor (BF). The *BF*_*10*_ describes the ratio between the evidence for the hypothesis *H*_*1*_ relative to another hypothesis *H*_*0*_, where the latter typically is the null hypothesis. For example, when using a Bayesian *t*-test, *BF*_*10*_ = 5 denotes that the data are five times more likely under the hypothesis *H*_*1*_ (i.e. that there is an effect) than under *H*_*0*_ (i.e. no effect). Conversely, the BF_01_ describes the likelihood of the data under *H*_*0*_. The strength of evidence is interpreted as follows: *BF*_*10*_ < 1, no evidence; *BF*_*10*_ = 1–3, anecdotal evidence for *H*_*1*_; *BF*_*10*_ = 3–10, moderate evidence for *H*_*1*_; *BF*_*10*_ = 10–30, strong evidence for *H*_*1*_; *BF*_*10*_ = 30–100, very strong evidence for *H*_*1*_; and *BF*_*10*_ > 100, extreme evidence for *H*_*1*._ The equivalent applies when reporting *BF*_*01*_ for *H*_*0*_.

We chose to use Bayesian statistics instead of traditional frequentist *t*-tests and ANOVAs because Bayesian statistics can give the strength of evidence for the null hypothesis, which frequentist tests do not provide. In addition, using Bayesian statistic, we are provided with richer information on the difference of means and standard deviations, the influence of outliers, and power of the test.

## Results

### First trial gaze shift latency

A Bayesian ANOVAs revealed no indication of group differences in gaze shift latencies of the first trial at the different time points. Instead, there was moderate evidence for the null hypothesis (i.e. no group differences) at 10 and 14 months of age. At 18 months, we found anecdotal evidence for the null hypothesis.

Regarding developmental change, there was moderate evidence for the null hypothesis in a Bayesian ANOVA. The finding suggests that infants with a subsequent diagnosis of ASD did not differ from neurotypically developing infants in terms of the change of first trial gaze shift latency across ages. In addition, we found anecdotal evidence for the null hypothesis regarding the average across ages, which further indicates that the gaze shift latencies on the first trial were similar in all groups (see Table 2 for all descriptive statistics, as well as Bayes Factors, and Fig. 2 for visualization of descriptive statistics).

**Table 2:** Descriptive statistics of the dependent variables displayed for each group (*n*, *Mean*, *SD*) and Bayesian statistics (*BF*_01,_ moderate evidence for the null hypothesis is marked with *)

	LL (29, 14 females)			EL-no-ASD (47, 31 females)			EL-ASD (14, 6 females)			Bayesian ANOVA
	*n*	*Mean*	*SD*	*n*	*Mean*	*SD*	*n*	*Mean*	*SD*	*BF*_*01*_^*a, b*^
*First trial, gaze shift latency*										
At 10 m	21	301.4	145.4	28	267.1	108.6	8	256.7	139.6	4.240*
At 14 m	19	225.9	71.2	26	224.7	81.3	6	181.2	62.2	3.445*
At 18 m	15	186.2	77.5	28	208.7	90.7	12	160.5	74.7	2.338
Developmental change	21	− 15.8	36.3	31	− 5.3	21.1	10	− 10.1	20.5	3.347*
Average across ages	28	252.9	95.5	40	232.3	76.3	13	197.0	67.9	1.934
*Adaptation rates, gaze shift latency*										
At 10 m	25	− 2.120	16.07	36	− 8.259	13.83	9	− 4.790	10.71	2.420
At 14 m	22	− 3.787	9.80	33	− 0.954	13.17	7	− 4.648	16.41	4.513*
At 18 m	20	− 2.237	9.74	35	− 7.370	9.57	12	1.833	8.60	4.718^c^
Developmental change	25	− 0.206	2.82	37	− 0.324	1.78	10	0.521	2.17	5.109*
Average across ages	29	− 2.305	10.35	46	− 6.424	14.65	13	− 1.782	6.03	3.017*
*First trial, pupil response*										
At 10 m	25	98.4	5.9	36	97.8	8.1	9	96.2	7.3	5.933*
At 14 m	22	98.7	8.3	34	98.7	6.1	7	95.7	6.7	4.615*
At 18 m	20	100.7	5.0	37	102.0	6.0	12	99.7	5.9	4.007*
Developmental change	25	.2	1.3	38	0.4	1.2	10	.8	1.1	3.701*
Average across ages	29	99.3	5.3	48	99.4	6.0	13	97.1	5.1	4.664*
*Adaptation rates, pupil responses*										
At 10 m	25	0.016	0.32	36	0.023	0.30	9	0.008	0.16	7.156*
At 14 m	22	0.069	0.40	33	0.120	0.33	7	− 0.054	0.28	3.984*
At 18 m	20	− 0.128	0.28	35	− 0.023	0.37	12	0.042	0.37	3.524*
Developmental change	25	− 0.018	0.080	37	− 0.004	0.08	10	− 0.010	0.04	6.204*
Average across ages	29	0.029	0.24	48	− 0.004	0.22	12	− 0.019	0.24	6.998*

Gaze shift latency is displayed in msec, whereas pupil responses are displayed as change measure (in %) from pupil size after the occlusion passage relative to a baseline measure (i.e., 100%)

^a^*BF*_*01*_ describes the likelihood of the data under *H*_*0*_*, BF*_*01*_ = 1–3, anecdotal evidence for *H*_*0*_

^b^*BF*_*01*_ describes the likelihood of the data under *H*_*0,*_* BF*_*01*_ = 3–10, moderate evidence for *H*_*0*_

^c^This *BF* refers to the likelihood of the data under *H*_*1*_*, BF*_*10*_ = 3–10, moderate evidence for *H*_*1*_

### Adaptation rate of gaze shift latency

First, a Bayesian one sample *t*-test against 0 tested the average adaptation rate of gaze shift latency, irrespective of group and age. The result showed strong evidence for a decrease in gaze shift latency over trials (*BF*_*10*_ = 16.87, strong evidence for *H*_*1*_, *n* = 89, *M* =  − 4.33, *SD* = 12.39).

In a next step, Bayesian ANOVAs gave a similar result for the adaptation rate of gaze shift latencies at the different time points, with most support for the null hypothesis. We found anecdotal evidence for no group differences at 10 months of age and moderate evidence the null hypothesis at 14 months of age (see Table 2 for statistics of all tests). However, at 18 months of age, there was moderate evidence for the alternative hypothesis (*BF*_*01*_ = 4.718, see Table 2), as the EL-no-ASD group showed the highest adaptation rate and the EL-ASD group showed the lowest adaptation rate. Follow up analyses comparing pairs of groups showed moderate evidence for differences between the EL-no-ASD and EL-ASD groups, *BF*_*10*_ = 8.256, anecdotal evidence for differences between the EL-no-ASD and LL groups, *BF*_*10*_ = 1.214, and anecdotal evidence for the null hypothesis when comparing LL and EL-ASD, *BF*_*01*_ = 1.701.

Regarding developmental change, we found moderate evidence for no group differences in a Bayesian ANOVA. The finding indicates that infants in all groups showed similar change in adaptation rates of gaze shift latency across ages. Similarly, there was moderate support in favor of the null hypothesis regarding the average across ages (see Table 2 for statistics of all tests).

### First trial pupil response

Unexpectedly, testing the average across ages against the baseline value (100%) gave anecdotal evidence for the null hypothesis (*BF*_*01*_ = 2.382) suggesting that the first trial pupil response did not differ from baseline.

By using Bayesian ANOVAs, we tested group differences of the first trial pupil response at the different time points. The analyses showed moderate evidence for the null hypothesis at 10, 14 and 18 month of age.

**Fig. 3:**
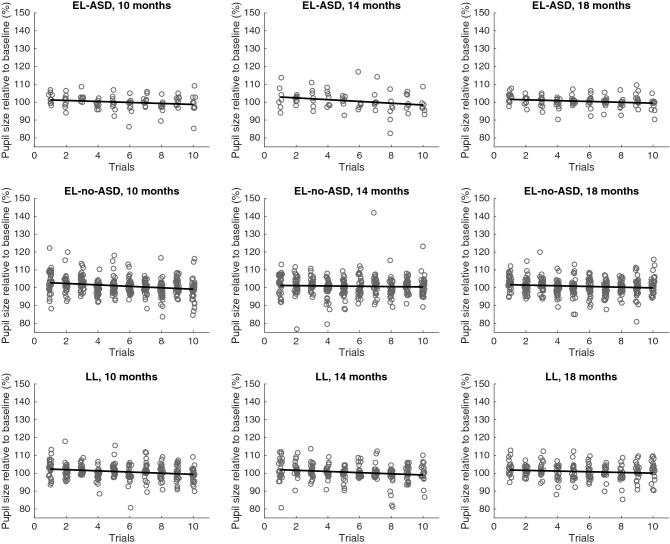
Change in pupil size after the gaze shift after temporary occlusion relative to a baseline measure over trials for all groups (EL-ASD, EL-no-ASD, LL) and ages. Circles represent individual trials for each infant

Similarly, the developmental change and the average across ages for the pupillary response on the first trial showed no indication for group differences (all *BF*s > 3, see Table 2 for all statistics, and Fig. 3 for visualization of descriptive statistics). These results suggest that there were no differences in first trial pupillary response between groups at the different time points, nor in terms of change in pupil response across ages.

### Adaptation rate of pupil response

First, a Bayesian one sample *t*-test against 0 tested the average adaptation rate of pupillary response, irrespective of group and age. The result indicated that the average adaptation rate of pupillary response was close to 0 (*BF*_*01*_ = 16.87, strong evidence for *H*_*0*_, *n* = 89, *M* =  − 0.03, *SD* = 0.03).

In a next step, we used Bayesian ANOVAs to test for group differences of the adaptation rates of pupillary responses at the different time points. The results showed moderate evidence for the null hypothesis at 10, 14 and 18 month of age.

Regarding developmental change and average across ages, the results were similar, providing moderate evidence for the null hypothesis (all *BF*s > 3, see Table 2 for all statistics) and suggesting that there were no differences between groups in terms of adaptation rates of pupillary responses.

### Additional analysis on pupil response

Given the surprising null effect for overall effect on pupil dilation on the first trial and the lack of overall adaptation, we used a Bayesian one sample *t*-test including the average pupillary response over the first two trials. We reasoned that comparing the two first trials could provide better experimental control than comparing the first trial with the preceding baseline, due to potential luminance differences during baseline and the test phase. The result indicated a decrease in pupillary response from the first to the second trial (*BF*_*10*_ = 21.59, strong evidence for *H*_*1*_, *N* = 89, *M* =  − 1.81, *SD* = 5.05). In addition, we found moderate support for the null hypothesis, suggesting that there were no group differences in this measure (*BF*_*01*_ = 7.88, moderate evidence for *H*_*0*_; EL-ASD *n* = 14, *M* =  − 2.14, *SD* = 4.86; EL-no-ASD *n* = 46, *M* =  − 1.51, *SD* = 5.77; LL *n* = 29, *M* =  − 2.11, *SD* = 3.92). This result provide evidence in favor of the view that indeed there is pupil dilation to the first unexpected object reappearance.

### Supporting analysis

To further investigate how previous experiences influence expectations in the three groups, we used a similar approach to analyze another event within the stimuli. Specifically, when the moving object in reversed direction (Fig. 1), observers make a catch up saccade to the object in order to continue tracking it. The developmental change of these gaze shift latencies was compared between groups. A Bayesian ANOVA supported the null hypothesis that there were no differences between groups whether in the first trial (*BF*_*01*_ = 6.37*,* moderate evidence for *H*_*0*_, see Table 3) nor the adaptation rate (*BF*_*01*_ = 7.30*,* moderate evidence for *H*_*0*_, see Table 3). The result implies similar developmental changes of gaze shift latency in all groups. Regarding the developmental change of pupillary responses, we used a Bayesian ANOVA and found anecdotal evidence for the null hypothesis in the first trial (*BF*_*01*_ = 2.68, see Table 3). However, regarding the adaptation of the pupillary responses, a Bayesian ANOVA showed anecdotal evidence for the alternative hypothesis alternative hypothesis (*BF*_*1o*_ = 1.53, see Table 3), as adaptation was highest in the EL-no-ASD group and lowest in the EL-ASD group. Follow up analyses comparing pairs of groups showed anecdotal evidence for differences between the EL-no-ASD and the EL-ASD group, *BF*_*10*_ = 1.498, anecdotal evidence for differences between the EL-ASD and the LL group, *BF*_*10*_ = 1. 131, and anecdotal evidence for the null hypothesis when comparing LL and EL-no-ASD groups, *BF*_*01*_ = 1.232. While there was some support for group differences, these differences were rather small and inconclusive. A Bayesian one-sample *t*-test against 0 revealed that the developmental change of pupillary responses after the direction reversal was no different than 0 (*BF*_*01*_ = 4.99*,* moderate evidence for *H*_*0*_).

**Table 3:** Descriptive statistics of the dependent variables displayed for each group (*n*, *Mean*, *SD*) and Bayesian statistics (moderate evidence for the null hypothesis is marked with *)

Developmental change	LL (29, 14 females)			EL-no-ASD (47, 31 females)			EL-ASD (14, 6 females)			Bayesian ANOVA
	*n*	*Mean*	*SD*	*n*	*Mean*	*SD*	*n*	*Mean*	*SD*	*BF*_01_
Gaze shift latency, first trial	23	− 1.16	14.53	33	0.81	17.41	9	− 0.41	10.90	6.37^a*^
Gaze shift latency, adaptation	26	− 0.06	1.69	38	0.17	2.86	11	0.17	1.36	7.30^a*^
Pupil response, first trial	26	0.04	0.71	41	− 0.25	0.87	12	0.08	0.77	2.68^b^
Pupil response, adaptation	26	− 0.01	0.07	38	0.05	0.18	11	− 0.06	0.10	1.53^c^

Gaze shift latency is displayed in msec, whereas pupil responses are displayed as change measure (in %) from pupil size after the occlusion passage relative to a baseline measure (i.e., 100%)

^a^*BF*_*01*_ describes the likelihood of the data under *H*_*0*_*, BF*_*01*_ = 3–10, moderate evidence for *H*_*0*_

^b^*BF*_*01*_ describes the likelihood of the data under *H*_*0*_*, BF*_*01*_ = 1–3, anecdotal evidence for *H*_*0*_

^c^This *BF* refers to the likelihood of the data under *H*_*1*_*, BF*_*10*_ = 3–10, moderate evidence for *H*_*1*_

## Discussion

This longitudinal study compared infants who subsequently were diagnosed with ASD and neurotypical infants when reacting to unexpected object motion, using gaze shift latencies and pupillary responses. Young infants can to a large extent control their eyes, and both pupil size and eye movements are easy to measure reliably. By using a visual motion paradigm including temporary occlusion, we examined expectations of the directionality of moving objects and how these expectations were updated with respect to new, incoming information. Our findings suggest that, at least within the context studied here, updating predictive models of object motion appears to be intact in ASD at an early age, as all infants regardless of clinical outcome showed similar first trial responses and adaptation rates over time, both in terms of gaze shift latencies and pupillary responses. Across the various measures, we mostly found moderate support for the null-hypothesis, using Bayesian statistics. The only exception was that the adaptation rate of gaze shift latency at 18 months showed moderate evidence for a difference between the EL-no-ASD and the EL-ASD groups. With respect to this, the first trial gaze shift latency was highest in the EL-no-ASD group (although still not different from the other groups), suggesting that the adaptation rate in this group may reflect high initial values. Nevertheless, we noted that the adaptation rate was positive in the EL-ASD group and negative in the two other groups at this age. A future direction for research could be to include older children, as differences in the adaptation rates of gaze shift latency may only be visible at a later age. Relatedly, we also noted that descriptively, the developmental change of gaze shift latency was positive in the EL-ASD group and negative in the two other groups; however, there is no statistical evidence for a difference between groups. Rather, the null hypothesis received moderate support.

In our visual motion task, the object moved along an unexpected trajectory, urging infants to update their predictions based on experiences from seeing the object on previous trials (Kochukhova and Gredeback 2007). Our results contribute to a long line of research on how infants learn to track and represent objects (Aguiar and Baillargeon 1999; Baillargeon 1999; Kochukhova and Gredeback 2007; Meltzoff and Moore 1998; Moore et al. 1978; Rosander and von Hofsten 2004; Spelke et al. 1994; von Hofsten et al. 2000). The ability to perceive stable objects that persist despite temporary occlusion develops early in life. Infant’s ability to track, maintain and even update predictions of object motion is critical from a developmental perspective as it forms what and how infants experience and perceive their environment (Kochukhova and Gredeback 2007; Rosander and von Hofsten 2004). The current study indicates that these developmental processes are robust to the atypicalities found in ASD. However, it should be noted that understanding object motion may be a less experience-dependent process compared with other forms of cognitive processing (Spelke et al. 1994), and future research should include different types of prediction tasks (including social ones) to more fully evaluate predictive abilities in ASD early in life.

Consistent with the eye movement data, we also did not observe any differences between groups in the pupillary responses following the occlusion event at any age, or averaged across ages. We expected to find a change in pupil size after the first trial due to the processing of unpredictable movement; however, the first trial pupil response was no different than the baseline measure. A previous developmental study from our group, which also included a subgroup of the infants assessed here, investigated the pupillary light reflex, and showed different developmental trajectories for the pupillary response in infants later diagnosed with ASD and infants who developed typically (Nyström et al. 2018). While both studies used the pupil size as the dependent measure, we expected that our pupil measure tapped into cognitive processes associated with alertness, surprise, and violation of expectation (Gredeback and Melinder 2010; Jackson and Sirois 2009; Laeng et al. 2012). It thus appears as if the differences in physiologically driven pupillary responses between groups, from our previous study, do not translate to cognitively mediated pupillary responses in infancy. However, there are other studies (see Blaser et al. 2014) that in fact found that found pupillary differences in cognitive tasks (i.e. visual search) between toddlers with and without ASD. Such findings suggest that differences in pupil size may arise due to differences in allocation of attention. In comparison, our stimuli were designed to elicit a surprise response, and did not require the prolonged cognitive processing required for visual search. Therefore, we do not consider our finding inconsistent with previous research using pupil dilation in young ASD populations.

The supporting analysis on gaze shift latencies and pupillary responses after the object reversed its direction showed a similar result as for the occlusion passage. Regarding the developmental change of gaze shift latency, we found no indication of group difference as the null hypothesis received moderate support. However, regarding the developmental change of pupillary response, there was anecdotal evidence for the alternative hypothesis, suggesting that the infants in the EL-ASD group showed a more negative slope compared to the other groups. It is worth to note that this result is inconclusive as the differences between groups were rather small and the change in pupil size after the direction reversal, in general, was close to 0.

Investigating prediction in relation to predictive coding theories of ASD would suggest alterations in how predictive models are formed and updated (Lawson et al. 2014; Pellicano and Burr 2012; Sinha et al. 2014; Van de Cruys et al. 2014). Our experiment was not designed to test the specific accounts mentioned earlier (it was implemented in our study in 2011, i.e. before these studies were published), but it is nevertheless tempting to argue that if the process of forming predictions was impaired in ASD (Pellicano and Burr 2012), the EL-ASD group should have reacted with less surprise to the unexpected motion pattern of the object. Specifically, due to broader priors about object motion, the pre-occlusion path would not be extrapolated as much as in the other groups, which could have resulted in a shorter gaze shift latency to the actual (unexpected) location of object reappearance on the first trial compared to the EL-noASD and LL group (because infants with ASD, less than other children, would have to inhibit the most dominant response—to extrapolate linear motion). In addition and for the same reason, in the EL-ASD group, the unexpected object motion should result in less pupil dilation. However, according to the alternative account put forward by Van de Cruys et al. (2014), if the precision of prediction error is set inadequately high, the EL-ASD group may show an increased pupillary response, and possibly a longer gaze shift latency on the first trial, compared the other groups. According to this view, updating expectation based on novel information would occur faster in the EL-ASD group. Thus, the EL-ASD group should have shown a steeper adaptation rate in both gaze shift latencies and pupillary responses compared with neurotypically developing infants.

Theoretically interesting as this discussion may be, our results indicate that infants with a subsequent diagnosis of ASD were no different than neurotypically developing infants in reacting to unexpected object motion and in updating their expectations in light of new information. Thus, we found no support for neither theory. Similarly, a study using a probabilistic learning task found that autistic children used the information available for the task no different than neurotypically developing children and adults (Manning et al. 2017).

In this study, we quantified gaze shifts latencies that spanned both what is considered predictive gaze shifts (in infants, approx. 200 ms and below; Engel et al. 1999; Falck-Ytter et al. 2006; Gredebäck et al. 2006) and reactive gaze shifts (200 ms and above). This was done to increase the amount of data available for data analysis. Thus, while we generally interpret the data in terms of predictive models of visual motion, we cannot claim that we exclusively studied prediction at the behavioral level. However, we note that a substantial amount of the gaze shifts, particularly for the older ages, were below 200 ms, and hence to be considered predictive in an absolute sense (see Fig. 2).

While we did not find evidence for early difference in predictive abilities in our study, we cannot exclude the possibility that other aspects of prediction may be atypical. As noted, previous studies have found both typical and atypical predictive abilities in autistic individuals (Croydon et al. 2017; Karaminis et al. 2016; Laeng et al. 2018; Lawson et al. 2015; Tewolde et al. 2018; Van de Cruys et al. 2017). Van de Cruys et al. (2014) suggested that prediction is atypical only in certain circumstances in ASD. One particular situation characterized by its uncertainty is the social situation. Relatedly, social atypicalities are amongst the most prominent features of ASD. For example, studying spontaneous eye movements, von Hofsten, Uhlig, Adell, and Kochukhova (2009) found indications that autistic children did not form predictions about the dynamics of a social interaction to the same extent as neurotypical children. Forming predictions in a social situation may be fundamentally different from predictions of non-social events as in our task.

A limitation of the current study is the small sample size. In our final sample the amount of children who received an ASD diagnosis at 36 months of age was 12, and even lower in longitudinal analyses where some participants had missing values. Thus, our results should be regarded as preliminary, as generalizability is related to sample size. While the relatively small sample size may challenge the statistical robustness, the Bayes factors are conclusive in most cases, showing clear age effects across groups and support for group similarities. Another reason to trust the results is that all trials were visually inspected, and noisy data that was hard to interpret was excluded. It is thus unlikely that the null results are due to noise in the measurements. A second limitation, regarding the stimulus, was that all infants saw the same stimulus in both blocks. In future studies, it would be favorable to include various object trajectories in order to test differential expectations about object motion. However, this was not possible in the current study due to time constraint in the larger longitudinal EASE project. Another aspect to consider for future studies is to investigate differential expectations about object motion related to vertical versus horizontal trials. While there might be differential expectations with respect to a “gravity” prior, we believe that this applies for both vertical as well as horizontal trials. A third limitation is that the stimulus presentation included a music track to increase attention to the stimuli. Adding music may have increased movement in some infants and caused noisy data. On the other hand, excluding music might have led to inattention and more missing data.

In conclusion, the findings of this study indicate that infants at elevated likelihood for ASD, regardless of clinical outcome, are able to adapt to unexpected object motion and that their performance was similar to that of neurotypical infants. Forming and updating predictions with respect to novel information is crucial for day-to-day living skills, especially in the vulnerable period of early development when observing and learning from others is crucial. It has been suggested that learning about regularities in the environment and forming predictions about novel instances may be impaired in ASD. Our findings indicate that the ability to update representations about regularities in the world in light of new information may not function differently in infants with later ASD. However, this does not exclude the possibility that there are other aspects of prediction that are atypical in infants with subsequent ASD.

## Supplementary Information

Below is the link to the electronic supplementary material.

(PDF 647 kb)

(PDF 669 kb)
