# Supplementary figures and images for: Updating Expectations About Unexpected Object Motion in Infants Later Diagnosed with Autism Spectrum Disorder
Source: J Autism Dev Disord. 2021 Jan 30;51(11):4186–98. doi: 10.1007/s10803-021-04876-2 (PMC8510946; doi:10.1007/s10803-021-04876-2)

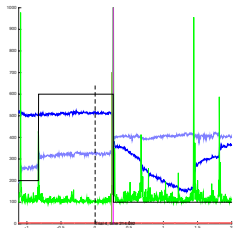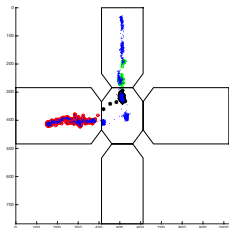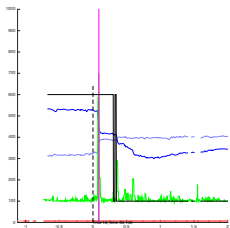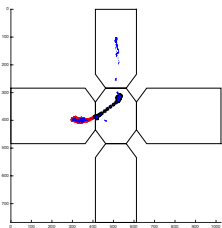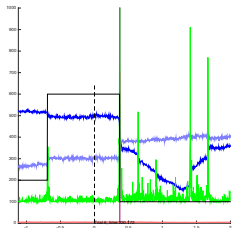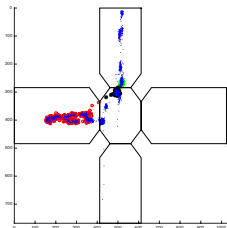

Supplement: Supplementary file 1 — (PDF 647 kb) [file 10803_2021_4876_MOESM1_ESM.pdf]

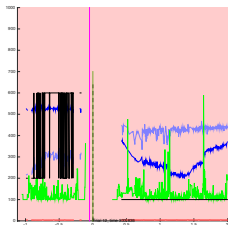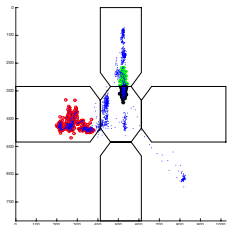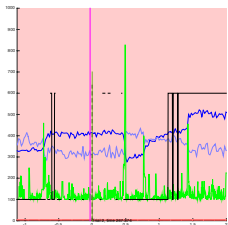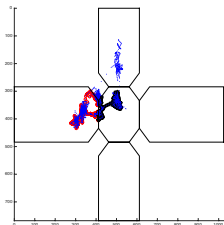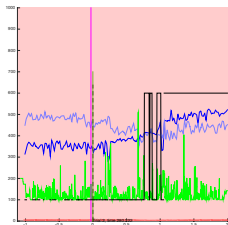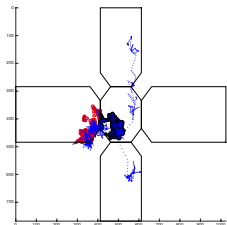

Supplement: Supplementary file 2 — (PDF 669 kb) [file 10803_2021_4876_MOESM2_ESM.pdf]
